# Energy Metabolic Reprogramming Characterized by Succinate Accumulation Aggravates Necrotizing Enterocolitis

**DOI:** 10.3390/ijms27198691

**Published:** 2026-09-29

**Authors:** Xiao-Lin Yan, Yu He, Xiao-Chen Liu, Yu-Ni Zhang, Fang-Ling Tang, Lei Bao, Yuan Shi

**Affiliations:** Department of Neonatology, Children’s Hospital of Chongqing Medical University, National Clinical Research Center for Children and Adolescents’ Health and Diseases, Ministry of Education Key Laboratory of Child Development and Disorders, Chongqing Key Laboratory of Child Rare Diseases in Infection and Immunity, Chongqing 400014, China; 2023140324@stu.cqmu.edu.cn (X.-L.Y.); heyu@hospital.cqmu.edu.cn (Y.H.); 2023130290@stu.cqmu.edu.cn (X.-C.L.); 2023140325@stu.cqmu.edu.cn (Y.-N.Z.); 2023111088@stu.cqmu.edu.cn (F.-L.T.)

**Keywords:** necrotizing enterocolitis, energy metabolism, succinate, HIF-1α/IL-1β signaling pathway, succinate dehydrogenase

## Abstract

Necrotizing enterocolitis (NEC) is a severe inflammatory intestinal disorder of preterm infants associated with substantial morbidity and mortality. This study investigated energy metabolic alterations in NEC and the contribution of succinate to disease progression. Fecal energy metabolites were quantified by high-performance liquid chromatography–mass spectrometry in preterm infants and neonatal mice. Neonatal C57BL/6 mice were assigned to the Control, NEC, NEC+dimethyl malonate (DMM), NEC+succinate (Suc), and NEC+DMM+Suc groups. Ileal tissues were analyzed by metabolomics, transcriptomics, histopathology, immunofluorescence, flow cytometry, Western blotting, reverse transcription quantitative PCR, and succinate dehydrogenase (SDH) activity assays. Both human neonates and neonatal mice with NEC exhibited metabolic reprogramming characterized by prominent succinate accumulation. Exogenous succinate further altered intestinal metabolism, reduced survival, aggravated weight loss and intestinal injury, decreased tight junction protein expression and lamina propria CD4^+^Foxp3^+^ regulatory T-cell proportions, and enhanced hypoxia-inducible factor-1α (HIF-1α)/interleukin-1β (IL-1β) signaling. Intestinal SDH activity and succinate dehydrogenase complex flavoprotein subunit A (SDHA) expression were increased in NEC and further enhanced by exogenous succinate, accompanied by increased colocalization of SDHA with F4/80^+^ macrophages. Pharmacological inhibition of SDH with DMM partially improved survival, intestinal injury, barrier disruption, and Treg loss, while suppressing HIF-1α/IL-1β signaling. These findings identify succinate accumulation as a metabolic hallmark of NEC and support a potential succinate–SDH–HIF-1α/IL-1β axis linking metabolic dysregulation to intestinal injury and immune imbalance.

## 1. Introduction

Necrotizing enterocolitis (NEC) is a life-threatening inflammatory bowel disorder of preterm infants, characterized by rapid onset, fulminant progression, and high mortality [1,2,3]. Despite its complex and multifactorial pathogenesis, accumulating evidence indicates that it is closely associated with immature intestinal barrier development, excessive innate immune activation, insufficient establishment of adaptive immune tolerance, and gut microbiota-associated metabolic dysregulation [4,5,6,7]. However, the precise molecular mechanisms linking these processes remain incompletely understood. In recent years, increasing attention has been directed toward the role of immunometabolic imbalance in NEC pathogenesis [4,6,8,9].

Given the immaturity of the intestinal barrier in preterm infants, abnormal colonization by commensal or opportunistic pathogenic bacteria may alter the local metabolic milieu, including short-chain fatty acids, bile acids and secondary bile acids, and tryptophan-derived metabolites [10,11,12]. These bioactive metabolites may directly impair intestinal barrier homeostasis and drive inflammatory responses through modulation of tight junction protein expression, cellular metabolic reprogramming, and immune cell function [6]. In particular, increasing interest has focused on the crosstalk between energy metabolism and intestinal barrier function [13]. Short-chain fatty acids derived from the gut microbiota serve as important energy sources for intestinal epithelial cells and are essential for maintaining metabolic homeostasis [13,14], whereas metabolic disturbances in intestinal tissues may compromise epithelial barrier integrity and impair mucosal immune cell function [14,15,16]. Thus, disruption of intestinal metabolic homeostasis may represent a critical mechanistic bridge linking barrier injury to uncontrolled intestinal inflammation in NEC.

Among the metabolites implicated in immunometabolic regulation, succinate has attracted particular attention in recent years [17,18,19,20]. Succinate, an essential intermediate of the tricarboxylic acid (TCA) cycle, has emerged as a pivotal metabolic signal bridging microbial metabolism, host bioenergetics, and inflammatory immune pathways [17,21]. Aberrant succinate accumulation not only reflects disturbances in central carbon metabolism but also acts as a pro-inflammatory signaling metabolite [21,22]. Accumulating evidence indicates that succinate may stabilize hypoxia-inducible factor-1α (HIF-1α) through inhibition of prolyl hydroxylase activity or promotion of mitochondrial reactive oxygen species production associated with succinate dehydrogenase (SDH) activity, thereby enhancing the expression of proinflammatory mediators, including interleukin-1β (IL-1β) [23,24]. Through these mechanisms, succinate may amplify local inflammatory responses and contribute to tissue injury.

In our previous study, we demonstrated that microbiota-associated succinate levels are significantly elevated in NEC and are associated with disease progression. These effects may involve alterations in macrophage activation states and activation of the succinate receptor 1 (SUCNR1)/HIF-1α signaling axis [25]. However, a comprehensive understanding of succinate-centered metabolic reprogramming in NEC remains lacking, particularly regarding its potential impact on other immune cell populations, including regulatory T cells, and the underlying molecular mechanisms remain to be elucidated.

Furthermore, SDH, a critical metabolic hub connecting succinate accumulation with cellular functional remodeling, has emerged as a potential therapeutic target. Dimethyl malonate (DMM), a classical SDH inhibitor, has been widely investigated in experimental models of inflammatory diseases [24,26,27]. Nevertheless, whether succinate accumulation represents a characteristic metabolic signature of NEC and whether targeting succinate metabolism can modulate disease progression remains largely unknown.

To address these questions, we characterized the energy metabolic profiles of fecal and intestinal tissue samples from infants with NEC and neonatal mouse models and identified succinate as a major differential metabolite. We further investigated the effects of succinate on NEC progression and explored the underlying mechanisms, with particular focus on SDH-dependent inflammatory signaling. These findings may offer mechanistic insights into the metabolic underpinnings of NEC and highlight potential targets for therapeutic intervention.

## 2. Results

### 2.1. Baseline Clinical Characteristics of Enrolled Neonates

This study included 12 neonates with NEC and 12 matched non-NEC controls. Baseline clinical characteristics are shown in Table 1. These baseline demographic and clinical data were derived from the same previously established clinical cohort [25]. In the present analysis, the same dataset was reused for a different analytical purpose, specifically to evaluate the association between clinical infection-related indicators and metabolic alterations. No significant differences were observed between the two groups in sex, gestational age, postnatal age, corrected gestational age, birth weight, or 1 min Apgar score.

Comparison of inflammatory indices showed that serum procalcitonin (PCT) levels were significantly higher in neonates with NEC than in controls [8.72 (1.03, 13.38) ng/mL vs. 0.687 (0.186, 1.234) ng/mL, *p* = 0.0023]. White blood cell count, neutrophil percentage, and platelet count did not differ significantly between the two groups. Detailed results are shown in Table 1.

### 2.2. Fecal Energy Metabolic Profiling in Infants with NEC

To explore fecal energy metabolic disturbances in NEC, we examined the differences in fecal energy metabolic intermediates between NEC and matched non-NEC neonates. Key intermediates of the TCA cycle are illustrated in Figure 1a. Further analysis showed that, compared to controls, neonates with NEC exhibited significantly increased fecal concentrations of succinate and L-malate. Consistent with our previous study [25], fecal succinate concentrations were 1878.0 ± 1711 nmol/g in the NEC group and 313.0 ± 530.3 nmol/g in the control group (*p* = 0.009), whereas fecal L-malate concentrations were 277.4 ± 205.3 nmol/g and 101.2 ± 90.64 nmol/g, respectively (*p* = 0.0244).

The levels of α-ketoglutarate, oxaloacetate, lactate, nicotinamide adenine dinucleotide (NAD), fumarate, and pyruvate showed upward trends in the NEC group, although the differences did not reach statistical significance. In contrast, cis-aconitate and citrate showed downward trends, but these differences were also not statistically significant (Figure 1b). These findings indicate that preterm infants with NEC exhibited an altered fecal energy metabolic profile, characterized predominantly by a marked increase in succinate.

We next analyzed the correlations between fecal succinate levels and birth characteristics or inflammatory indices. No significant correlations were observed between fecal succinate levels and gestational age, postnatal age, birth weight, or 1 min Apgar score in either the NEC or control group (Figure 1c). In the NEC group, fecal succinate levels showed a moderate positive correlation with white blood cell count (r = 0.613, *p* = 0.034) and a moderate negative correlation with serum PCT levels (r = −0.594, *p* = 0.042) but were not significantly correlated with neutrophil percentage or platelet count. In the control group, fecal succinate levels were not significantly correlated with white blood cell count, neutrophil percentage, platelet count, or PCT levels (Figure 1d).

### 2.3. Fecal Energy Metabolic Profiling in Neonatal Mice with NEC and Succinate Intervention

To characterize fecal energy metabolic profiles in neonatal mice with NEC and exogenous succinate intervention, we compared fecal energy metabolic intermediates among different groups. Compared to the control group, the NEC group exhibited significant increases in most measured intermediates, with the exception of cis-aconitate. Fecal succinate was significantly elevated in NEC mice (1847.0 ± 630.7 vs. 453.5 ± 486.3 μmol/g, *p* = 0.0001), consistent with our previous findings [25]. Exogenous succinate further increased fecal succinate levels in the NEC+Suc group (4796.0 ± 2502 μmol/g vs. NEC, *p* = 0.0136; Figure 2a), without significantly altering other metabolic intermediates. In addition to succinate, the NEC+Suc group showed non-significant trends toward increased α-ketoglutarate and lactate levels, and decreased oxaloacetate, L-malate, cis-aconitate, NAD, citrate, and fumarate compared with the NEC group (Figure 2b–i). Overall, NEC was characterized by a broad disturbance in fecal energy metabolism, whereas exogenous succinate selectively exacerbated succinate accumulation without substantially altering the overall metabolic profile.

To determine whether oral succinate supplementation was accompanied by increased systemic succinate availability, we additionally measured serum succinate concentrations in neonatal mice. Serum succinate concentrations were 659.2 ± 174.2, 999.7 ± 319.7, and 1614 ± 480.8 μmol/L in the Control, NEC, and NEC+Suc groups, respectively, with significant increases in NEC versus Control (*p* = 0.0414) and NEC+Suc versus NEC (*p* = 0.0003; Supplementary Figure S1).

### 2.4. Exogenous Succinate Promotes Disease Aggravation, Intestinal Damage, and Reduction in Regulatory T Cells in Neonatal Mice with NEC

To assess the effect of exogenous succinate on NEC progression, disease severity was evaluated in neonatal mice following succinate supplementation. Kaplan–Meier analysis showed significantly lower survival in the NEC group than in the Control group, with a further reduction in the NEC+Suc group relative to the NEC group (log-rank test, *p* < 0.05; Figure 3a). During the experimental period, Control mice exhibited normal weight gain, whereas body weight declined in the NEC group and decreased further in the NEC+Suc group (Figure 3b). At the end of NEC induction, body weights were 5.24 ± 0.42 g, 3.58 ± 0.33 g, and 2.95 ± 0.24 g in the Control, NEC, and NEC+Suc groups, respectively, with significant differences among groups (Figure 3c). These results indicate that exogenous succinate further aggravates weight loss and reduces survival in neonatal mice with NEC.

Histopathological examination revealed varying degrees of intestinal injury in the NEC and NEC+Suc groups, including villous edema, disrupted villous architecture, partial villous sloughing, thinning of the muscular layer, and separation of the mucosal, submucosal, and muscular layers. In contrast, intestinal architecture remained intact in the Control group (Figure 3d) Histological injury scores were 0.50 (0.00, 1.00), 2.00 (2.00, 2.75), and 3.50 (3.00, 4.00) in the Control, NEC, and NEC+Suc groups, respectively, with significant differences among groups (Figure 3e). These results indicate that exogenous succinate further exacerbated NEC-associated intestinal injury.

Double-immunofluorescence staining showed that the mean fluorescence intensities of Occludin and ZO-1 were significantly lower in the NEC group than in the Control group and were further reduced in the NEC+Suc group relative to the NEC group (Figure 3f–h). These findings suggest that exogenous succinate further impairs intestinal tight junction protein expression and mucosal barrier integrity.

Flow cytometric analysis further showed that the proportion of CD4^+^Foxp3^+^ T cells in the intestinal lamina propria was significantly lower in the NEC group than in the Control group (*p* = 0.0446) and decreased further in the NEC+Suc group compared with the NEC group (*p* = 0.0136) (Figure 3i,j). These results indicate that exogenous succinate further reduces intestinal Treg proportions in neonatal mice with NEC.

### 2.5. Exogenous Succinate Further Alters the Intestinal Energy Metabolic Profile in Neonatal Mice with NEC

To characterize intestinal energy metabolic alterations in neonatal mice with NEC and following succinate supplementation, intestinal energy metabolites were compared among the Control, NEC, and NEC+Suc groups. Principal component analysis (PCA) showed clear separation among the three groups, with samples within each group clustering closely, indicating distinct intestinal metabolic profiles (Figure 4a). KEGG pathway analysis showed that the altered metabolites were mainly associated with pathways related to energy metabolism, carbohydrate metabolism, amino acid metabolism, and digestive system functions (Figure 4b).

Analysis of key energy metabolic intermediates showed that intestinal succinate and α-ketoglutarate levels were significantly higher in the NEC group than in the Control group (Figure 4c). Succinate concentrations were 62.40 ± 7.91 ng/mL in the NEC group and 33.57 ± 2.79 ng/mL in the Control group (*p* = 0.0261). In contrast, itaconate, lactate, L-glutamine, and L-glutamate showed decreasing trends, although these differences were not statistically significant. No significant changes were observed in the other measured central carbon metabolism-related metabolites.

Following exogenous succinate supplementation, intestinal succinate, α-ketoglutarate, and L-glutamate levels tended to increase further in the NEC+Suc group compared with the NEC group, although these differences did not reach statistical significance. Succinate concentrations were 598.00 ± 329.80 ng/mL and 62.40 ± 7.91 ng/mL in the NEC+Suc and NEC groups, respectively. In addition, fumarate, cis-aconitate, L-malate, lactate, pyruvate, and citrate levels were significantly increased in the NEC+Suc group relative to the NEC group, whereas itaconate showed a nonsignificant downward trend. Collectively, these findings indicate that intestinal energy metabolism is markedly altered during NEC and is further remodeled by exogenous succinate supplementation.

### 2.6. Exogenous Succinate Further Enhances HIF-1 Pathway Activation in Neonatal Mice with NEC

To investigate the molecular pathways associated with NEC progression and the effects of exogenous succinate, transcriptomic profiling was performed on intestinal tissues from the Control, NEC, and NEC+Suc groups. Principal component analysis (PCA) showed clear separation among the three groups, with samples within each group clustering closely, indicating distinct global gene expression profiles (Figure 5a). Venn diagram analysis further demonstrated both shared and group-specific gene expression patterns among the Control, NEC, and NEC+Suc groups (Figure 5b). These findings indicate substantial transcriptional remodeling during NEC, with additional changes following succinate supplementation.

KEGG enrichment analysis was then performed on genes commonly upregulated in the NEC and NEC+Suc groups and on genes uniquely upregulated in the NEC+Suc group. Commonly upregulated genes were predominantly enriched in the HIF-1 signaling pathway and related biological pathways (Figure 5c), whereas genes uniquely upregulated in the NEC+Suc group were mainly enriched in the HIF-1 signaling pathway and pathways associated with central carbon metabolism (Figure 5d). Gene set enrichment analysis (GSEA) of the whole-transcriptome dataset further showed significant enrichment of HIF-1 signaling in the NEC group relative to the Control group, with stronger enrichment in the NEC+Suc group compared with the NEC group (Figure 5e,f). Collectively, these findings suggest that HIF-1 signaling is activated during NEC and may be further enhanced by exogenous succinate, potentially contributing to succinate-associated aggravation of disease severity.

### 2.7. Exogenous Succinate Further Increases Intestinal SDHA Expression and SDH Activity in Neonatal Mice with NEC

Previous studies have linked SDH-dependent succinate oxidation to HIF-1α stabilization -mediated inflammation [24,28]; therefore, we further examined intestinal SDH activity and SDHA expression in NEC and succinate-supplemented neonatal mice.

Transcriptomic analysis showed that SDHA, SDHB, SDHC, and SDHD were upregulated in the NEC group compared with the Control group. In the NEC+Suc group, SDHA and SDHD remained elevated relative to controls but were lower than in the NEC group (Figure 6a). RT-qPCR was subsequently performed to validate the expression of these four genes. Among them, SDHA mRNA expression was significantly increased in the NEC group compared with the Control group and was further elevated in the NEC+Suc group relative to the NEC group (Figure 6b). Western blot analysis showed a similar pattern at the protein level, with SDHA expression increased in the NEC group and further enhanced following succinate supplementation (Figure 6c,d).

SDH activity was also significantly higher in the NEC group than in the Control group and was further increased in the NEC+Suc group compared to the NEC group (Figure 6e). To further characterize the distribution of SDHA within intestinal tissues and macrophages, double-immunofluorescence staining for SDHA and F4/80 was performed. SDHA fluorescence intensity was markedly increased in the NEC group relative to controls and was further enhanced in the NEC+Suc group (Figure 6f,g). Colocalization analysis further showed increased colocalization of SDHA with F4/80^+^ macrophages in the intestinal lamina propria in the NEC group, with a further increase following succinate supplementation.

Collectively, intestinal SDH activity and SDHA expression were increased during NEC and further enhanced by exogenous succinate.

### 2.8. DMM Alleviates NEC Severity and Suppresses Activation of the HIF-1α/IL-1β Signaling Axis

We further assessed whether DMM could alleviate NEC severity and counteract the aggravating effects of exogenous succinate. Kaplan–Meier analysis showed significantly higher survival in the NEC+DMM group than in the NEC group and in the NEC+DMM+Suc group than in the NEC+Suc group (log-rank test, *p* < 0.05; Figure 7a). Consistently, body weight loss during NEC induction was attenuated in both DMM-intervention groups. At the end of NEC induction, body weights were 5.46 ± 0.37 g, 3.41 ± 0.21 g, 3.72 ± 0.41 g, 2.80 ± 0.11 g, and 3.03 ± 0.14 g in the Control, NEC, NEC+DMM, NEC+Suc, and NEC+DMM+Suc groups, respectively, with significant differences among groups (Figure 7b,c). These findings suggest that DMM partially improves survival and attenuates body weight loss associated with NEC and exogenous succinate supplementation.

Histopathological examination showed reduced intestinal injury in the NEC+DMM group compared to the NEC group and in the NEC+DMM+Suc group compared with the NEC+Suc group. Histological injury scores were 0.00 (0.00, 0.00), 2.00 (2.00, 3.00), 1.00 (1.00, 1.00), 4.00 (3.00, 4.00), and 2.50 (2.00, 3.00) in the Control, NEC, NEC+DMM, NEC+Suc, and NEC+DMM+Suc groups, respectively, with significant differences among groups (Figure 7d,e). These results indicate that DMM partially alleviates NEC-associated and succinate-aggravated intestinal histopathological injury.

Double-immunofluorescence staining further showed that the mean fluorescence intensities of Occludin and ZO-1 were significantly higher in the NEC+DMM group than in the NEC group and were similarly increased in the NEC+DMM+Suc group relative to the NEC+Suc group (Figure 7f–h). These findings suggest that DMM partially restores tight junction protein expression and helps preserve intestinal mucosal barrier integrity.

Flow cytometric analysis showed that the proportion of CD4^+^Foxp3^+^ T cells in the intestinal lamina propria was significantly higher in the NEC+DMM group than in the NEC group (*p* = 0.0024) and was also increased in the NEC+DMM+Suc group compared to the NEC+Suc group (*p* = 0.0139) (Figure 7i,j). These results indicate that DMM partially reverses the reduction in intestinal Treg proportions associated with NEC and further aggravated by exogenous succinate.

To further assess the effect of DMM on HIF-1α/IL-1β signaling, intestinal HIF-1α and IL-1β protein expression were examined by Western blotting. Both HIF-1α and IL-1β levels were significantly lower in the NEC+DMM group than in the NEC group and were similarly reduced in the NEC+DMM+Suc group compared with the NEC+Suc group (Figure 7k–m). These findings indicate that DMM partially attenuates HIF-1α/IL-1β signaling in intestinal tissues during NEC, including under exogenous succinate supplementation.

## 3. Discussion

Proper energy metabolism is critical for maintaining core cellular processes in intestinal tissues, such as growth, proliferation, and differentiation [29,30]. NEC is a life-threatening gastrointestinal disease that predominantly occurs in preterm infants and continues to be a major contributor to morbidity and mortality [31]. In recent years, intestinal immunometabolic dysregulation has attracted increasing attention in the pathogenesis and progression of NEC [32,33], although the precise molecular mechanisms remain incompletely understood. In this study, we demonstrate that NEC is characterized by profound energy metabolic reprogramming, with succinate accumulation emerging as a central metabolic feature. Exogenous succinate further exacerbated intestinal injury, reduced Treg proportions, and aggravated disease severity, suggesting that succinate is not merely a metabolic byproduct but an active immunometabolic regulator in NEC. This study integrates previously published [25] and newly generated multi-omics datasets, providing a comprehensive framework to investigate succinate-associated immunometabolic reprogramming in NEC.

In clinical samples, we initially observed significantly elevated fecal levels of succinate and L-malate in infants with NEC, whereas α-ketoglutarate, oxaloacetate, lactate, NAD, fumarate, and pyruvate showed a consistent upward trend, while cis-aconitate and citrate tended to decrease. These findings suggest marked energy metabolic remodeling in infants with NEC. Based on metabolic pathway features, this pattern may be related to enhanced glutaminolysis, with carbon flux entering the TCA cycle mainly at the level of α-ketoglutarate and thereby promoting the generation of downstream metabolites [34]. By contrast, the decreased levels of citrate and cis-aconitate may be associated with insufficient acetyl-CoA supply, reduced activity of upstream enzymes such as citrate synthase or aconitase, or enhanced export of mitochondrial citrate into the cytosol [35,36]. Further analyses showed that fecal succinate was not significantly correlated with baseline birth characteristics but was associated with selected inflammatory indices. Notably, fecal succinate was negatively correlated with PCT in infants with NEC. This unexpected association should be interpreted cautiously given the exploratory nature and small sample size of the clinical cohort. PCT primarily reflects systemic inflammatory responses, whereas fecal succinate may more closely reflect the local intestinal metabolic environment. Moreover, although both measurements were obtained around the time of NEC diagnosis, blood and fecal sampling were not strictly synchronized in all infants, and this temporal mismatch may have contributed to the observed association. In addition, neonatal PCT levels can be influenced by postnatal age, physiological elevation, and perinatal stress [37,38,39]. Thus, these markers may not change in parallel during NEC progression. The relationship between fecal succinate and disease severity may also be dynamic or nonlinear, however, the limited sample size precluded a sufficiently powered Bell stage-stratified analysis. Larger prospective cohorts with synchronized blood and fecal sampling are needed to clarify these relationships.

In the animal experiments, we further confirmed that neonatal mice with NEC exhibited marked abnormalities in fecal energy metabolic profiles, among which succinate showed the most prominent elevation. After exogenous succinate supplementation, only fecal succinate levels were further increased, whereas no substantial changes were observed in the remaining terminal metabolites, suggesting that exogenous succinate did not broadly remodel the luminal end-metabolite spectrum. Meanwhile, exogenous succinate further aggravated body weight loss, mortality risk, and histopathological injury in neonatal mice with NEC, consistent with our previous findings [25,40]. We also found that exogenous succinate further reduced the expression of the tight junction proteins Occludin and ZO-1 in intestinal tissues. Given that loss of tight junction proteins is closely associated with intestinal barrier disruption in NEC [41,42,43], and that metabolic imbalance and oxidative stress may further impair tight junction structure and barrier function [13,44], our findings suggest that elevated succinate may worsen NEC by aggravating local metabolic stress and mucosal barrier injury.

Beyond barrier injury, we found that exogenous succinate further decreased the proportion of CD4^+^Foxp3^+^ T cells in the intestinal lamina propria of neonatal mice with NEC. Tregs play a pivotal role in maintaining intestinal immune tolerance and mucosal homeostasis, and reduced Treg proportions have been reported in both infants with NEC and experimental NEC models [45,46,47,48]. Previous studies have indicated that Treg homeostasis is tightly regulated by metabolic and oxidative stress cues [49,50,51]. Metabolites such as α-ketoglutarate can shape the fate of naïve CD4^+^ T cells by influencing mitochondrial metabolism and epigenetic programming [52], and TCA cycle activity, together with the succinate/α-ketoglutarate balance, is essential for T-cell proliferation and epigenetic regulation [53]. In addition, succinate has been shown to promote intestinal inflammation by accelerating Foxp3 degradation [54]. Collectively, these findings, together with our results, suggest that succinate accumulation in NEC may represent not only a consequence of metabolic dysregulation but also an active metabolic signal that impairs Treg maintenance and thereby contributes to intestinal immune imbalance.

At the intestinal tissue level, we found that succinate and α-ketoglutarate were significantly elevated in neonatal mice with NEC, indicating substantial local energy metabolic remodeling. Following exogenous succinate supplementation, levels of fumarate, cis-aconitate, L-malate, lactate, pyruvate, and citrate were further increased, whereas succinate itself only showed an upward trend without reaching statistical significance, which may be attributable to the small sample size and relatively high within-group variability. Together, these findings suggest that marked metabolic remodeling has already occurred in intestinal tissues during NEC, and that exogenous succinate can further exacerbate this process. Notably, serum succinate levels were also elevated in NEC mice and further increased following oral succinate supplementation, supporting increased systemic succinate availability under these experimental conditions. Thus, the effects of exogenous succinate may involve both local intestinal and systemic metabolic responses rather than broad remodeling of the fecal metabolite profile. However, the present data do not distinguish whether the increase in circulating succinate primarily reflects direct intestinal absorption or secondary alterations in intestinal, microbial, and host metabolism, and the relative contributions of local and systemic succinate exposure remain to be determined.

At the mechanistic level, transcriptomic analysis identified HIF-1 signaling as a prominent pathway associated with NEC and further enhanced by exogenous succinate. Increasing evidence indicates that intestinal metabolites and HIF-1 signaling are closely interconnected in the regulation of epithelial barrier integrity, immunometabolic reprogramming, and mucosal inflammation [18,55,56]. HIF-1α may also link metabolic alterations to adaptive immune dysregulation by modulating T-cell fate, particularly Treg differentiation, stability, and suppressive function in a context-dependent manner [57]. Consistent with this concept, our previous study implicated the SUCNR1/HIF-1α axis in succinate-mediated NEC progression [25]. SDH-dependent succinate oxidation has also been shown to promote mitochondrial ROS production, HIF-1α stabilization, and IL-1β expression in inflammatory macrophages [24], suggesting that SDH may represent an important metabolic link between succinate accumulation and inflammatory signaling.

In the present study, intestinal SDH activity and SDHA expression were increased during NEC and further enhanced by succinate supplementation. Notably, although bulk RNA-seq showed reduced expression of SDH subunits in the NEC+Suc group compared to the NEC group, targeted RT-qPCR, Western blotting, and immunofluorescence consistently demonstrated increased SDHA expression following succinate supplementation. This discrepancy may reflect methodological and biological variability between global transcriptomic profiling and targeted measurements, although its precise basis remains unclear. Importantly, the corresponding increase in SDH enzymatic activity provides direct functional evidence supporting enhanced SDH activity, therefore, our interpretation of SDH alterations is primarily based on targeted SDHA validation and direct enzymatic activity measurements rather than on transcript levels of individual SDH subunits. Moreover, increased colocalization of SDHA with F4/80^+^ macrophages suggests that macrophages may represent one relevant cellular compartment. However, our whole-tissue analyses cannot establish macrophages as the predominant source of SDH–HIF-1α/IL-1β activation or exclude contributions from other intestinal cell populations. Further cell-specific studies are required to clarify this issue.

Importantly, pharmacological inhibition of SDH using DMM significantly attenuated NEC severity. DMM improved survival, reduced intestinal injury, restored tight junction protein expression, and partially recovered Treg proportions, while suppressing HIF-1α/IL-1β signaling. Together, these findings suggest that SDH activity contributes to succinate-associated metabolic and inflammatory dysregulation in NEC and that its inhibition can partially counteract these pathological changes. Protective effects of DMM have also been reported in cardiac injury, neuroinflammation, and metabolic dysfunction-associated liver disease [26,28,58], where the modulation of mitochondrial metabolism and inflammatory signaling has been implicated. By inhibiting SDH-dependent succinate metabolism, DMM may reduce mitochondrial ROS generation and downstream inflammatory responses. SDH inhibition may also influence Treg-associated immune balance through broader metabolic reprogramming; however, the relevant cellular targets and mechanisms underlying these effects in NEC remain to be clarified.

Several limitations should be acknowledged. First, the relatively small clinical cohort may limit statistical power and generalizability, and the observed clinical associations do not establish causality. Second, although increased colocalization of SDHA with F4/80^+^ macrophages was observed, the cell-type-specific contribution of SDHA to NEC progression has not been functionally established, and contributions from intestinal epithelial and other cell populations cannot be excluded. Future studies incorporating larger clinical cohorts, cell-type-specific SDHA genetic models, primary-cell experiments, and rescue approaches will be required to define the cellular contribution of the succinate–SDH–HIF-1α axis to NEC pathogenesis and intestinal immune regulation.

## 4. Materials and Methods

### 4.1. Patients and Sample Collection

Between April and November 2021, a prospective cohort of neonates diagnosed with NEC stage II or higher, according to the modified Bell staging criteria, was enrolled from the Department of Neonatology, Children’s Hospital of Chongqing Medical University. NEC was diagnosed based on clinical symptoms, radiographic findings, and laboratory results in accordance with the modified Bell criteria [59,60]. Controls were recruited from neonates with non-intestinal diseases hospitalized during the same study period at a 1:1 individually matched ratio. Matching was performed based on gestational age at birth, postnatal age at sampling, and corrected gestational age (≤2-week difference), to minimize confounding related to developmental stage and sampling timing. A total of 24 neonates were included in the final cohort, comprising 12 NEC cases and 12 matched controls. This study included a predefined subset of a previously described cohort, which was independently selected for the present metabolomic analysis with a distinct analytical objective. Exclusion criteria included congenital intestinal malformations, guardian refusal, probiotic use before sample collection, and insufficient sample volume. Clinical data collected included sex, gestational age, age at enrollment, corrected gestational age, birth weight, 1 min Apgar score, white blood cell count, platelet count, neutrophil percentage, and procalcitonin concentration. The recruitment process is shown in Supplementary Figure S2. Laboratory parameters, including white blood cell count, platelet count, neutrophil percentage, and PCT, were obtained from routine blood tests performed around the time of NEC diagnosis. Fecal samples were collected at the time of clinical NEC diagnosis or shortly thereafter when spontaneous stool was unavailable. In such cases, samples were obtained by gentle sterile rectal stimulation to ensure adequate collection at the disease-relevant time point. Fresh fecal specimens were immediately transferred into sterile containers, transported on ice, aliquoted into two sterile 2 mL microcentrifuge tubes, and stored at −80 °C until further analysis. This study involving human participants was reviewed and approved by the Ethics Committee of Children’s Hospital of Chongqing Medical University (Approval No. 2021.23; approval date: 28 January 2021). Written informed consent was obtained from the legal guardians of all enrolled neonates.

### 4.2. Animal Experiments and Study Design

Specific pathogen-free (SPF) C57BL/6 mice were obtained from the Experimental Animal Center of Chongqing Medical University and housed at the Experimental Animal Center of Children’s Hospital of Chongqing Medical University under standard conditions (20–25 °C, 12 h light/dark cycle) with free access to food and water. Breeding pairs were maintained at a female-to-male ratio of 2:1, and pups were delivered naturally. All neonatal mice used in this study were derived from three independent experimental batches using different litters. To minimize maternal and litter-specific confounding effects, pups were allocated to experimental groups using litter-stratified randomization, with each litter contributing animals to all experimental groups. Random allocation was performed after birth and before experimental intervention using a computer-generated random sequence. At postnatal day 7, neonatal mice of either sex were assigned to five groups: Control, NEC, NEC+Suc, NEC+DMM, and NEC+DMM+Suc. NEC was induced by formula feeding combined with hypoxia and cold stress for four consecutive days. The hyperosmolar formula was prepared by dissolving 2 g Esbilac puppy milk replacer and 3.3 g Similac Advance infant formula in 10 mL drinking water or 1% succinate solution. Pups were gavage-fed at 0.03 mL/g body weight every 4 h starting at 08:00 daily. Twice daily between feedings, pups were exposed to 100% N_2_ for 60 s followed by cold stress at 4 °C for 10 min. DMM was administered intraperitoneally at 100 mg/kg once daily at 08:00 throughout the NEC induction period. During the experiment, body weight was recorded daily before the first feeding, and clinical manifestations, including diarrhea, abdominal distension, hematochezia, and mortality, were monitored. A total of 25 animals per group were included in the survival analysis at baseline. Animals that died during the experimental period were recorded as events at the time of death and were not included in body weight analyses at subsequent time points. Following euthanasia, intestinal luminal contents were gently collected before tissue harvesting, and intestinal tissues were subsequently collected for downstream molecular and biochemical analyses. Predefined subsets of animals were used for individual assays, with corresponding sample sizes provided in the figure legends. Histological evaluation and pathological scoring were performed in a blinded manner. Humane endpoints were predefined, and animals exhibiting severe distress were euthanized according to institutional guidelines. All animal procedures were approved by the Institutional Animal Care and Use Committee of Chongqing Medical University (Approval No. CHCCMU-IACUC20210316004; approval date: 16 March 2021). and conducted in accordance with institutional and national guidelines for the care and use of laboratory animals.

### 4.3. Morphological Examination and Histological Analysis

Terminal ileal tissues (approximately 1–2 cm) were collected from mice, fixed in 4% paraformaldehyde for 1–3 days, dehydrated, and paraffin-embedded. Sections (4 μm) were then deparaffinized, rehydrated, and subjected to H&E staining. A pathologist blinded to the experimental groups evaluated histopathological injury using a previously described NEC injury scoring system. Histological injury was graded as follows: grade 0, normal histology without injury; grade 1, mild injury with slight separation of the submucosa and/or lamina propria; grade 2, moderate injury characterized by moderate separation of the submucosa and/or lamina propria, with or without edema involving the submucosa and muscularis; grade 3, severe injury characterized by marked separation of the submucosa and/or lamina propria and/or pronounced edema of the submucosa and muscularis, together with focal villous sloughing; and grade 4, transmural necrosis with complete villous loss.

### 4.4. Double-Immunofluorescence Staining of Intestinal Tissues

Paraffin-embedded intestinal sections were deparaffinized, rehydrated, and subjected to antigen retrieval in citrate buffer (pH 6.0). After quenching endogenous peroxidase activity and blocking nonspecific binding with 3% bovine serum albumin, sections were incubated overnight at 4 °C with primary antibodies against Occludin (HUABIO, China, ET1701-76, 1:500), ZO-1 (HUABIO, China, ER41204, 1:1250), SDHA (Servicebio, China, GB153278, 1:2500), or F4/80 (Servicebio, China, GB113373, 1:500), followed by incubation with appropriate secondary antibodies and fluorescent dyes. For double-immunofluorescence staining, heat-mediated antibody stripping was performed after the first staining cycle before the second round of staining. Nuclei were counterstained with DAPI.

Occludin and ZO-1 images were acquired at ×400 magnification using a multichannel fluorescence scanner (Meca Scientific, China), with mean fluorescence intensity calculated from three randomly selected fields in each of three independent biological samples per group. F4/80 and SDHA images were acquired using a Nikon CSU-W1 spinning-disk confocal microscope (Nikon, Japan) equipped with a ×40 objective to assess antigen expression and spatial colocalization within intestinal tissues. Data were analyzed using one-way ANOVA followed by Tukey’s post hoc test in GraphPad Prism 10.0.3, with *p* < 0.05 considered statistically significant.

### 4.5. Flow Cytometric Analysis

Fresh intestinal tissues were harvested, and the surrounding mesenteric tissues were carefully removed. The intestines were opened longitudinally and thoroughly rinsed with pre-cooled RPMI-1640 medium to remove residual blood and luminal contents. Tissues were first incubated in Hank’s balanced salt solution containing 1 mmol/L EDTA, 1 mmol/L DTT, and 10% FBS at 37 °C for 30 min with shaking. After washing with cold RPMI-1640 medium, the tissues were minced and enzymatically digested in RPMI-1640 supplemented with 1 mg/mL collagenase VI, 1000 U/mL DNase I, and 10% FBS at 37 °C for 30 min with shaking. Digestion was terminated by adding an equal volume of RPMI-1640 medium, and the resulting cell suspension was filtered through a 70 μm cell strainer. After centrifugation and washing, single-cell suspensions were prepared for flow cytometric analysis.

Cells were first stained with Horizon™ Fixable Viability Stain 780 (BD Pharmingen, USA, 565388, 1:1000) at room temperature for 15 min in the dark. Surface staining was then performed with antibodies against CD45 (PerCP-Cy5.5 Rat Anti-Mouse CD45, BD Pharmingen, USA, 550994, 1:200), CD3e (FITC Hamster Anti-Mouse CD3e, BD Pharmingen, USA, 553061, 1:200), and CD4 (APC Rat Anti-Mouse CD4, BD Pharmingen, USA, 553051, 1:200) at 4 °C for 30 min in the dark. After fixation and permeabilization with Fix/Perm buffer, cells were incubated overnight at 4 °C with anti-Foxp3 antibody (PE Rat Anti-Mouse Foxp3, BD Pharmingen, USA, 563101, 1:200) for intracellular staining. Cells were subsequently resuspended in staining buffer and subjected to flow cytometric analysis to determine the proportion of CD45^+^CD3^+^CD4^+^Foxp3^+^ cells. The detailed gating strategy is shown in Supplementary Figure S3.

### 4.6. Metabolite Determination

A portion of the fecal metabolomic data analyzed in this study was derived from previously generated datasets and reanalyzed for the present study with a distinct mechanistic objective. Specifically, fecal succinate levels were reported in our previous publication [25], whereas additional fecal energy metabolites derived from the same cohort were further analyzed in the current study to investigate metabolic reprogramming associated with NEC. By contrast, intestinal tissue metabolomic data from neonatal NEC mice were newly generated in this study and have not been reported previously. Fecal energy metabolites were quantified using previously validated high-performance liquid chromatography–mass spectrometry (HPLC–MS) methods [25], and all samples were processed under identical analytical conditions to ensure inter-sample comparability.

Targeted metabolomic profiling of intestinal tissues was performed using ultra-high-performance liquid chromatography coupled with electrospray ionization tandem mass spectrometry (UHPLC–ESI–MS/MS) on a QTRAP mass spectrometry platform. Briefly, 106 reference standards of energy metabolites were prepared in methanol and serially diluted in 50% methanol to generate calibration curves. Approximately 50 mg of intestinal tissue was homogenized in 500 μL of 80% methanol using cryogenic grinding, followed by ultrasonic extraction. After centrifugation at 4 °C (14,000× *g*, 15 min), supernatants were either directly mixed with internal standards or dried under nitrogen and reconstituted in 50% acetonitrile containing internal standards, depending on metabolite requirements. Chromatographic separation was performed using an ExionLC AD system (AB Sciex, USA) at 40 °C on either a BEH HILIC column (5 μm, 4.6 × 250 mm) or an Agilent Eclipse XDB-C18 column (5 μm, 4.6 × 150 mm). Mobile phases for HILIC consisted of ammonium bicarbonate/ammonia buffer and acetonitrile, while reversed-phase separation used 0.2% acetic acid in water and methanol. Mass spectrometric detection was conducted using AB SCIEX QTRAP 7500 or QTRAP 6500 systems operated in positive and/or negative ion modes. Data acquisition and quantification were performed using SCIEX OS software (version 4.0, SCIEX, Framingham, MA, USA). Peak integration was performed automatically and manually verified when necessary. Metabolite concentrations were determined using linear regression calibration curves.

### 4.7. Serum Succinate Measurement

At the end of NEC induction, blood samples were collected from neonatal mice via orbital bleeding and centrifuged at 3000 rpm for 15 min at 4 °C to obtain serum. Serum succinate concentrations were measured using an Amplex Red Succinate Assay Kit (Beyotime Biotechnology, China, S0529S) according to the manufacturer’s instructions. The colorimetric assay was used, and absorbance at 570 nm was measured using a BioTek Synergy H1 microplate reader (Agilent Technologies, Winooski, VT, USA). Succinate concentrations were calculated from a standard curve generated using the succinate standards provided with the kit.

### 4.8. Transcriptome Profiling of Intestinal Tissues

Intestinal tissues were pulverized under liquid nitrogen, and total RNA was subsequently isolated using TRIzol reagent. RNA yield and purity were assessed with a NanoDrop 2000 spectrophotometer (Thermo Fisher Scientific, USA). RNA integrity was initially assessed by agarose gel electrophoresis and further evaluated using an Agilent 5300 Fragment Analyzer (Agilent Technologies, USA). Qualified RNA samples were subjected to poly(A) mRNA enrichment using oligo(dT)-attached magnetic beads, followed by RNA fragmentation, first- and second-strand cDNA synthesis, end repair, A-tailing, adaptor ligation, size selection, and PCR amplification for library construction. Library concentration was quantified using a Qubit 4.0 fluorometer (Thermo Fisher Scientific, USA), and sequencing was performed on the NovaSeq X Plus platform (Illumina, USA). Raw sequencing data were subjected to quality control by excluding reads containing adaptor contamination, low-quality sequences, excessive ambiguous bases, or insufficient length, thereby generating clean reads for subsequent analysis. Clean reads were aligned to the reference genome, and gene/transcript abundance was estimated using RSEM. Differentially expressed genes (DEGs) were identified using DESeq2, with FDR < 0.05 and |log2 fold change| ≥ 1 as the significance thresholds. KEGG enrichment analysis of DEGs was performed using Fisher’s exact test followed by Benjamini–Hochberg correction for multiple testing, with adjusted *p* < 0.05 considered statistically significant.

### 4.9. Quantitative Real-Time PCR

Total RNA was extracted from mouse intestinal tissues using a *SteadyPure* RNA Extraction Kit (Accurate Biology, China, AG21024) according to the manufacturer’s instructions, and RNA concentration was measured with a NanoDrop 2000 spectrophotometer (Thermo Fisher Scientific, USA). Equal amounts of RNA were reverse-transcribed into cDNA with an *Evo M-MLV* RT Premix Kit (Accurate Biology, China, AG11734). RT-qPCR was performed on a Bio-Rad CFX real-time PCR system (Bio-Rad, Hercules, CA, USA) using SYBR Green *Pro Taq* HS Premix (Accurate Biology, China, AG11759). Relative mRNA expression was normalized to β-actin and calculated using the 2^−ΔΔCt^ method. Primer sequences are listed in Supplementary Table S2.

### 4.10. Western Blot Assessment

Total protein was extracted from intestinal tissues using RIPA buffer supplemented with protease inhibitor cocktail at a 100:1 (*v*/*v*) ratio. Frozen intestinal tissues were weighed and homogenized in 2 mL tubes containing magnetic beads and lysis buffer (100 μL per 10 mg tissue) using a tissue homogenizer for four cycles at 60 Hz for 30 s with 15 s intervals. After incubation on ice for 20–30 min, the lysates were centrifuged at 4 °C for 10 min, and the supernatants were collected. Protein concentrations were determined using a bicinchoninic acid (BCA) assay kit. Protein samples were mixed with 5× loading buffer at a 4:1 ratio, denatured at 100 °C for 10 min, and stored at −80 °C until use. Equal amounts of protein (50 μg per lane) were separated by SDS-PAGE on polyacrylamide gels prepared using a PAGE gel rapid preparation kit (Yeasen, China) and transferred onto 0.45 μm polyvinylidene fluoride (PVDF) membranes. After blocking for 30 min at room temperature, membranes were incubated overnight at 4 °C with primary antibodies against HIF-1α (GeneTex, USA, GTX127309, 1:1000), IL-1β (Abclonal, China, A22257, 1:2000), or SDHA (Servicebio, China, GB153278, 1:1000). After washing with TBST, membranes were incubated with HRP-conjugated goat anti-rabbit secondary antibody for 1 h at room temperature. Protein bands were visualized using enhanced chemiluminescence (ECL) reagents and captured with an iBright CL1500 imaging system (Thermo Fisher Scientific, USA). β-Actin was used as the loading control, and band intensities were quantified using ImageJ software (version 1.51j8, National Institutes of Health, Bethesda, MD, USA). Target protein expression was normalized to β-actin.

### 4.11. SDH Activity Assay

SDH activity in freshly collected mouse intestinal tissues was determined immediately using an assay kit (Solarbio, China, BC0955) according to the manufacturer’s instructions. Briefly, tissue lysate supernatants were mixed with the supplied reagents, and absorbance was measured at 600 nm using a BioTek Synergy H1 microplate reader. SDH activity was calculated according to the manufacturer’s protocol.

### 4.12. Statistical Analysis

All statistical analyses were performed using GraphPad Prism 10.0.3 (GraphPad Software, San Diego, CA, USA). Continuous variables are presented as mean ± standard deviation (SD) and analyzed using paired or unpaired *t*-tests, as appropriate, or one-way analysis of variance (ANOVA) followed by appropriate post hoc multiple comparison tests. Paired tests were applied for individually matched clinical comparisons. When the assumption of homogeneity of variance was not met, Brown–Forsythe and Welch ANOVA were applied. Non-normally distributed continuous variables or ordinal data were presented as median (interquartile range, IQR) and analyzed using the Kruskal–Wallis test followed by Dunn’s post hoc test. Categorical variables were compared using Fisher’s exact test. Survival outcomes were analyzed using Kaplan–Meier curves and compared using the log-rank (Mantel–Cox) test. Correlations between metabolite levels and clinical or inflammatory parameters were assessed using Spearman’s rank correlation. Transcriptomic analyses and figure generation were performed using the Majorbio Cloud Platform. All statistical tests were two-sided, and *p* < 0.05 was considered statistically significant.

## 5. Conclusions

In conclusion, our study demonstrates that NEC is associated with energy metabolic reprogramming characterized by elevated succinate. Increased succinate aggravates intestinal injury and is accompanied by a reduction in lamina propria Tregs, potentially contributing to NEC progression through the HIF-1α/IL-1β axis. Collectively, these findings support SDH as a promising therapeutic target and further advance our understanding of the metabolic mechanisms involved in NEC.

## Figures and Tables

**Figure 1:**
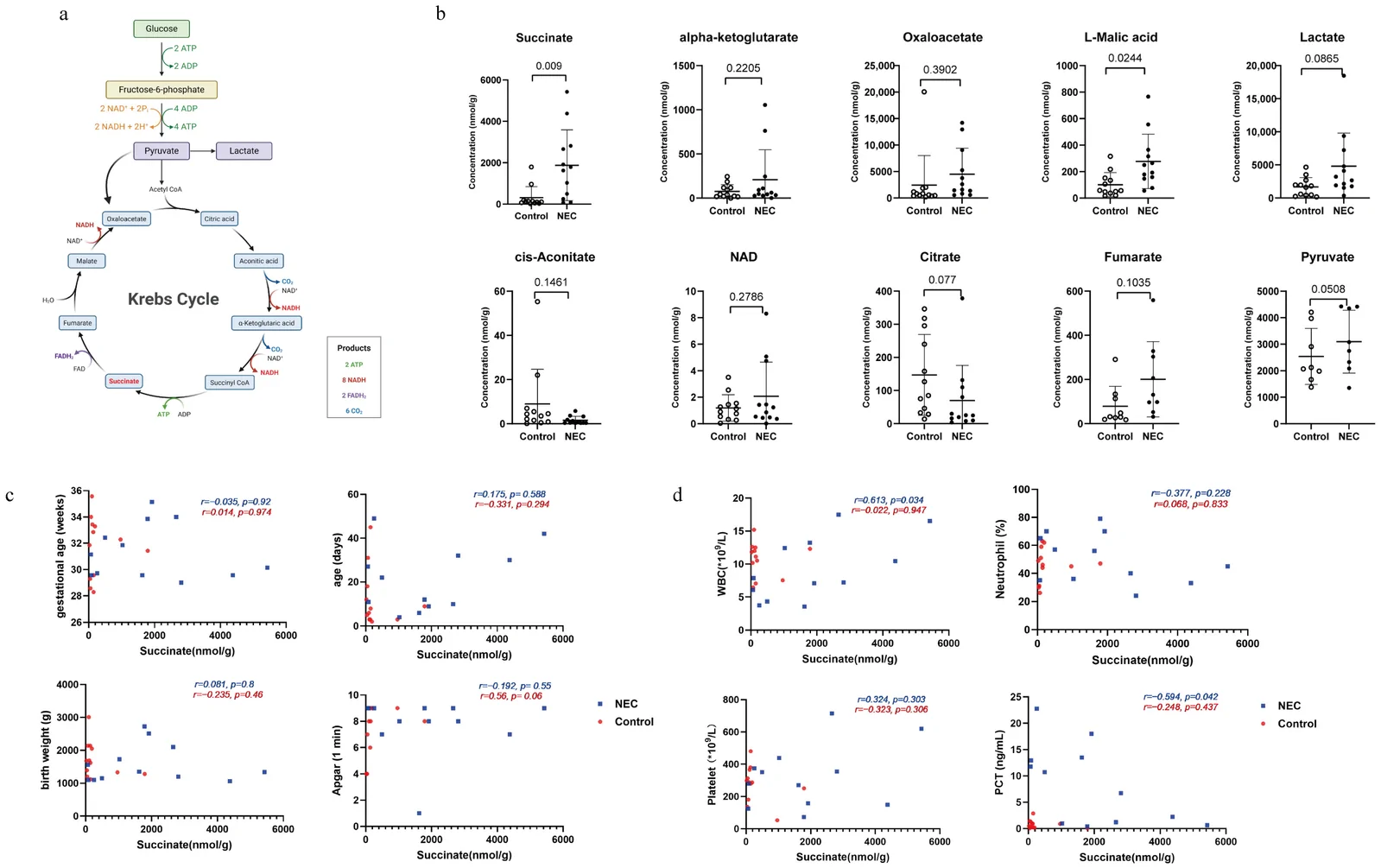
Fecal energy metabolic alterations and succinate accumulation in neonates with NEC. (**a**) Schematic illustration of the tricarboxylic acid (TCA) cycle. (**b**) Comparison of fecal energy metabolic intermediates between NEC and control groups. (**c**) Correlation analysis between fecal succinate levels and birth characteristics. (**d**) Correlation analysis between fecal succinate levels and inflammatory indices. Fecal succinate data were derived from a previously published cohort and re-analyzed for integrative analysis in the present study. Data are presented as mean ± SD (*n* = 12 per group). Statistical comparisons were performed using paired *t*-tests, and correlations were assessed using Spearman’s rank correlation test.

**Figure 2:**
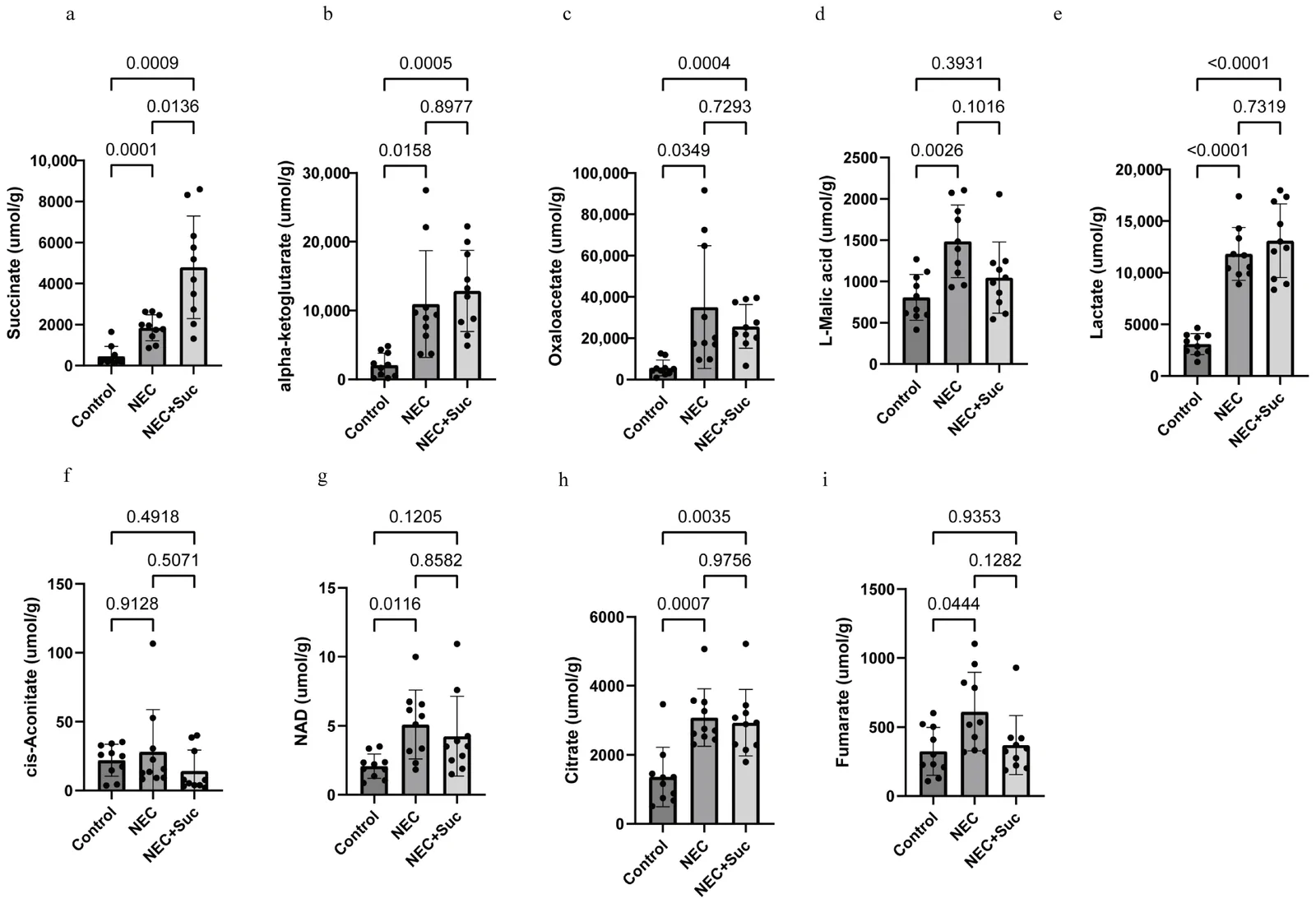
Fecal energy metabolic intermediates are altered in neonatal mice with NEC. (**a**–**i**) Fecal levels of succinate, α-ketoglutarate, oxaloacetate, L-malic acid, lactate, cis-aconitate, NAD, citrate, and fumarate. Fecal succinate data were derived from a previously published cohort and re-analyzed for integrative analysis in the present study. Data are presented as mean ± SD (*n* = 10 per group). Statistical analysis was performed using one-way ANOVA followed by Tukey’s multiple-comparison test.

**Figure 3:**
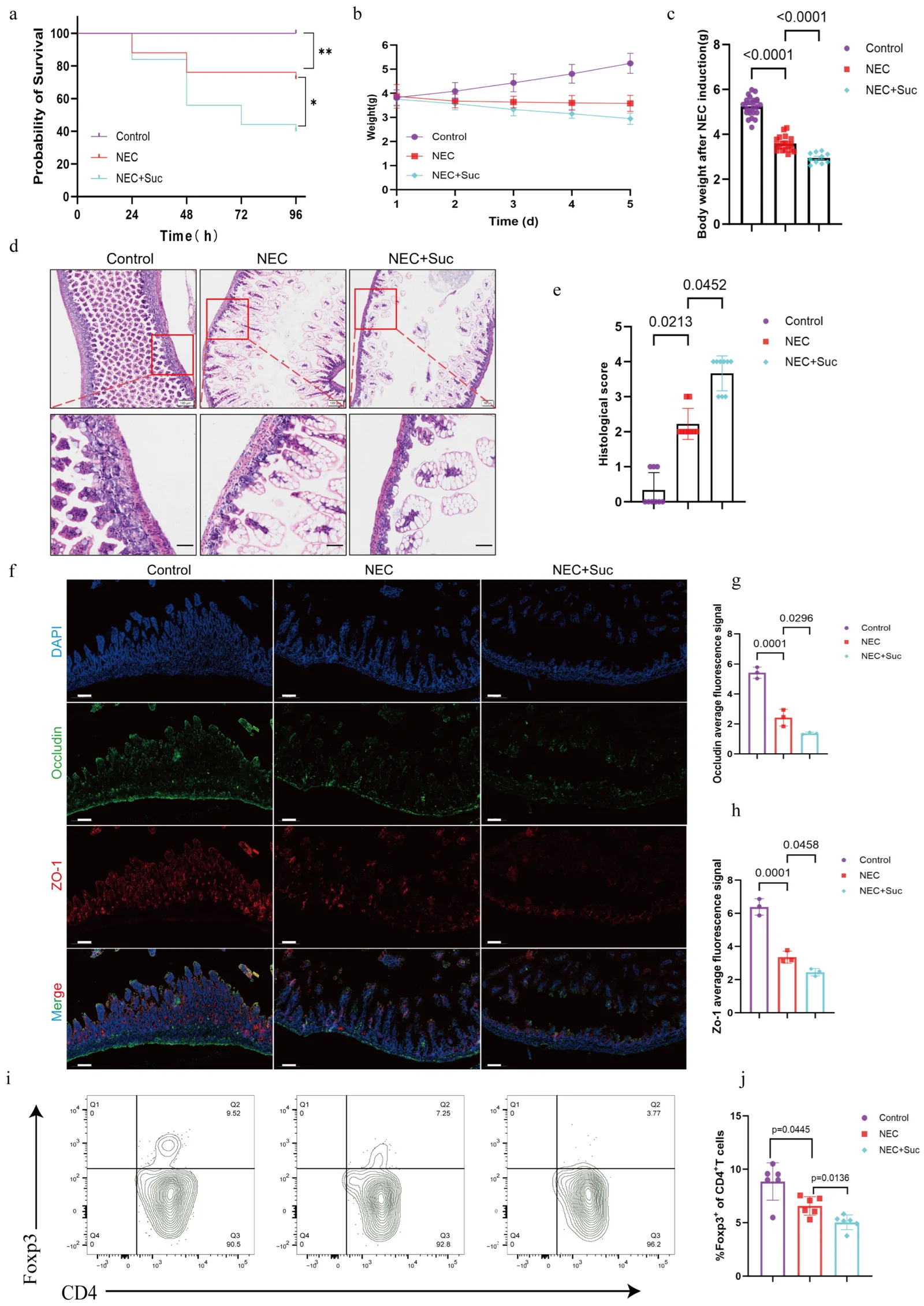
Exogenous succinate exacerbates disease severity in neonatal mice with NEC. (**a**) Kaplan–Meier survival curves (*n* = 25 per group). (**b**,**c**) Body weight curves and final body weight; numbers of animals at each time point are provided in Supplementary Table S1. (**d**) Representative H&E staining of terminal ileum (×200; scale bar = 100 μm). (**e**) Histological injury scores (*n* = 9 per group). (**f**) Representative immunofluorescence images of Occludin and ZO-1 (×400; scale bar = 100 μm). (**g**,**h**) Quantification of Occludin and ZO-1 mean fluorescence intensity (*n* = 3 per group); three random fields were averaged for each sample. (**i**) Representative flow cytometry plots of CD4^+^Foxp3^+^ T cells in lamina propria. (**j**) Quantification of CD4^+^Foxp3^+^ T cells (*n* = 6 per group). Data are presented as mean ± SD. Survival analysis was performed using the Kaplan–Meier method with log-rank test; other comparisons were analyzed using one-way ANOVA with appropriate post hoc tests. * *p* < 0.05; ** *p* < 0.01.

**Figure 4:**
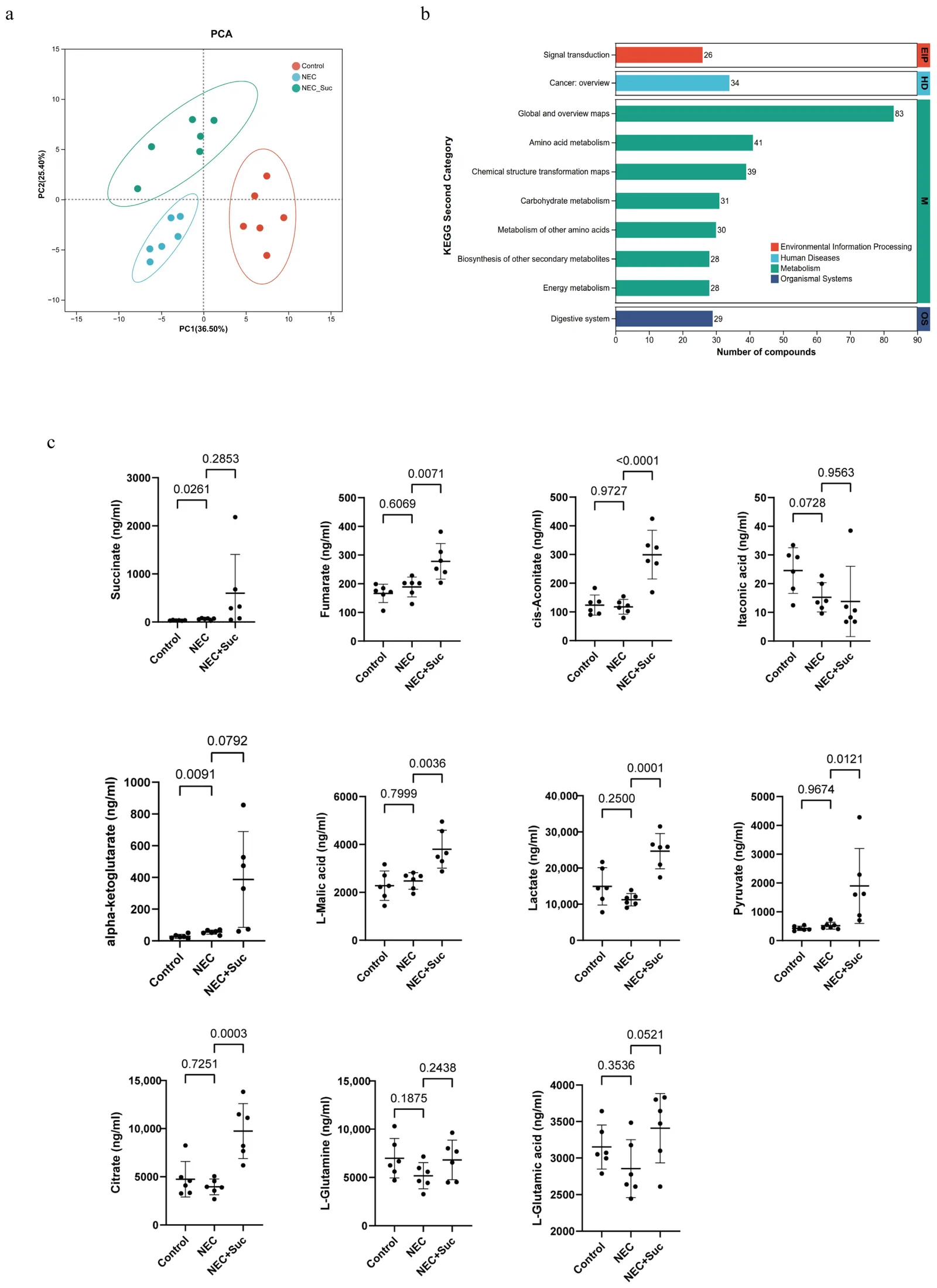
Altered intestinal energy metabolic intermediates in neonatal mice across groups. (**a**) Principal component analysis (PCA) of samples from each group. (**b**) KEGG pathway enrichment analysis of intestinal metabolites. (**c**) Comparison of intestinal metabolites, including succinate, fumarate, cis-aconitate, itaconate, α-ketoglutarate, L-malic acid, lactate, pyruvate, citrate, L-glutamine, and L-glutamic acid. Data are presented as mean ± SD (*n* = 6 per group). Statistical analysis was performed using one-way ANOVA followed by Tukey’s multiple-comparison test.

**Figure 5:**
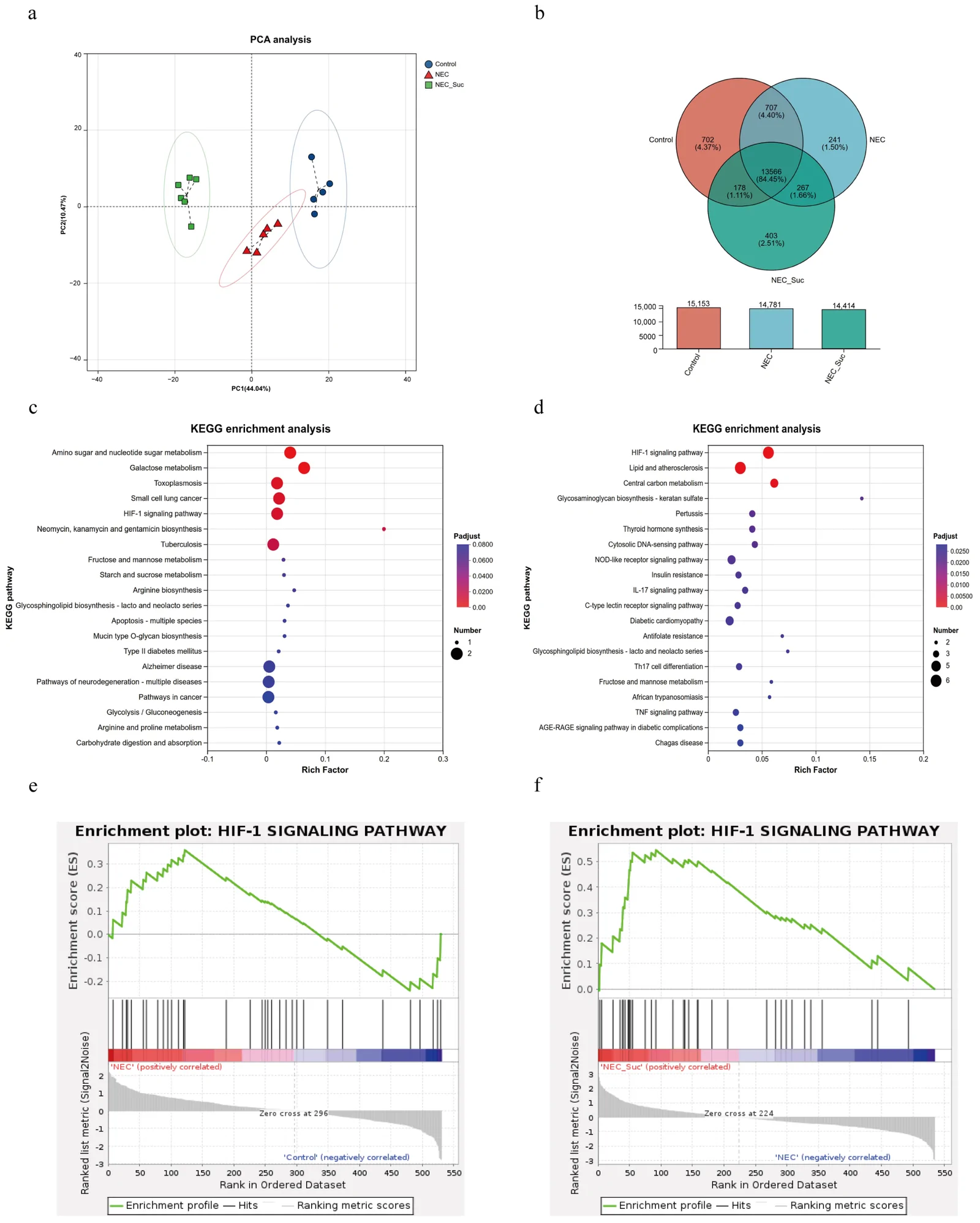
Exogenous succinate further enhances HIF-1 pathway activation in intestinal tissues of neonatal mice with NEC. (**a**) PCA of intestinal transcriptomic profiles. (**b**) Venn diagram of shared and group-specific expressed genes. (**c**) KEGG enrichment of genes commonly upregulated in the NEC and NEC+Suc groups. (**d**) KEGG enrichment of genes specifically upregulated in the NEC+Suc group. (**e**,**f**) GSEA of HIF-1 signaling in Control vs. NEC and NEC vs. NEC+Suc, respectively. Sample sizes: Control, *n* = 5; NEC, *n* = 5; NEC+Suc, *n* = 6.

**Figure 6:**
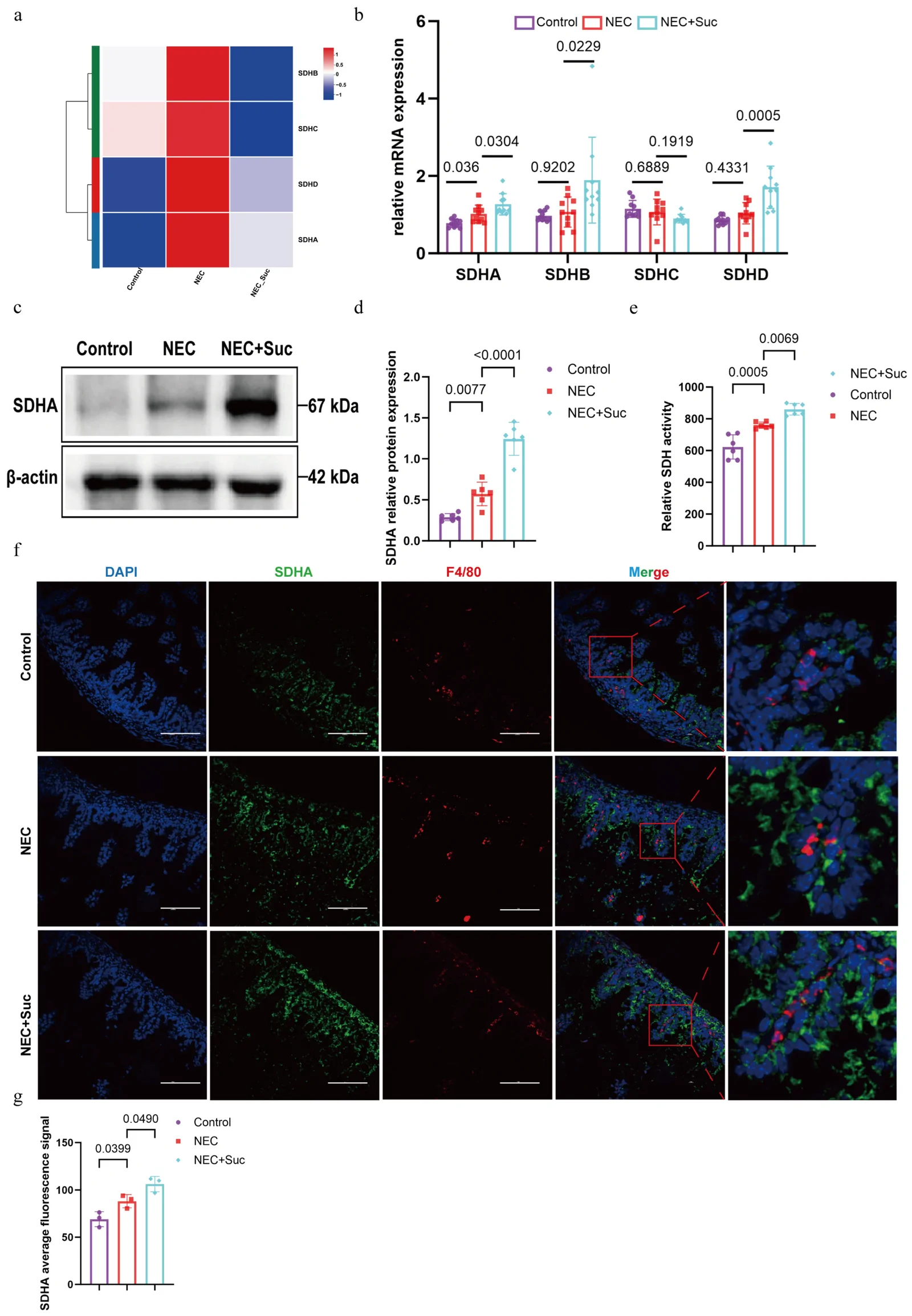
Succinate supplementation further increases SDHA expression and SDH activity in intestinal tissues of neonatal mice with NEC. (**a**) Heatmap of SDHA, SDHB, SDHC, and SDHD expression from transcriptomic data. (**b**) Relative mRNA expression of SDHA, SDHB, SDHC, and SDHD (*n* = 10 per group). (**c**) Representative Western blot of SDHA. (**d**) Densitometric analysis of SDHA protein expression (*n* = 6 per group). (**e**) SDH activity in intestinal tissues (*n* = 6 per group). (**f**) Representative immunofluorescence images of SDHA acquired with a ×40 objective; scale bar = 10 μm. (**g**) Quantification of SDHA mean fluorescence intensity (*n* = 3 per group); three random fields were averaged for each sample. Data are presented as mean ± SD and analyzed by one-way ANOVA followed by Tukey’s multiple-comparison test. *p* < 0.05 was considered statistically significant.

**Figure 7:**
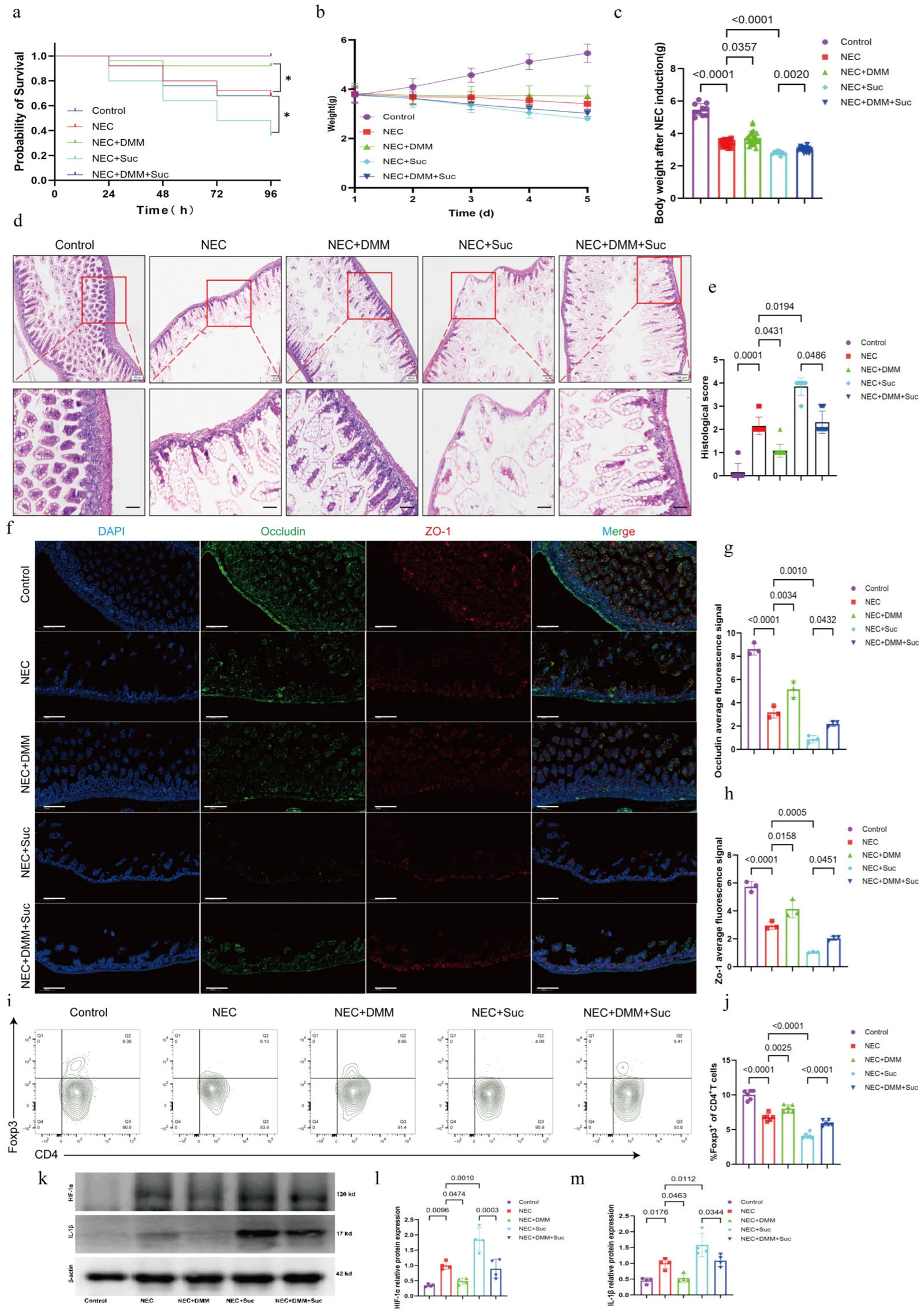
DMM alleviates NEC severity and succinate-induced injury via inhibition of the HIF-1α/IL-1β axis. (**a**) Kaplan–Meier survival curves (Control, *n* = 10; other groups, *n* = 25). (**b**,**c**) Body weight curves and final body weight; numbers of animals at each time point are provided in Supplementary Table S1. (**d**) Representative H&E staining of terminal ileum (×200; scale bar = 100 μm). (**e**) Histological injury scores (*n* = 13 per group). (**f**) Representative immunofluorescence im-ages of Occludin and ZO-1 (×400; scale bar = 100 μm). (**g**,**h**) Quantification of Occludin and ZO-1 mean fluorescence intensity (*n* = 3 per group); three random fields were averaged for each sample. (**i**) Representative flow cytometry plots of CD4^+^Foxp3^+^ T cells in lamina propria. (**j**) Quantification of CD4^+^Foxp3^+^ T cells (*n* = 6 per group). (**k**) Representative Western blots of HIF-1α and IL-1β. (**l**,**m**) Densitometric analysis of HIF-1α and IL-1β expression from four independent biological samples per group (*n* = 4). Data are presented as mean ± SD. Survival analysis was performed using the Kaplan–Meier method with log-rank test; other comparisons were analyzed using one-way ANOVA with appropriate post hoc tests. * *p* < 0.05.

**Table 1:** Patient characteristics.

Variable	Control (*n* = 12)	NEC (*n* = 12)	*p*-Value
**General Information**			
Female, % (n)	33.3 (4)	25(3)	1.0
Gestational age, $\bar{x}$ ± SD, w	31.7 ± 2.32	31.33 ± 2.1	0.687
Age at enrollment, M (IQR), d	7 (3–16.5)	17 (9.25–31.5)	0.052
CGA at enrollment, ± SD, w	33.43 ± 2.36	34.36 ± 2.09	0.318
Birth weight, $\bar{x}$ ± SD, g	1721 ± 541.3	1577 ± 575.3	0.535
Apgar 1 min, M (IQR)	8 (6.25–9)	8.5 (7.25–9)	0.630
**Laboratory Investigation**			
White cell count ×10^9^/L, M (IQR)	11.47 (8.19–12.46)	7.53 (4.78–13.04)	0.347
Neutrophil (%), $\bar{x}$ ± SD	46.08 ± 12.19	50.83 ± 17.72	0.452
Platelet Count ×10^9^/L, M (IQR)	287.5 (198.3–352.8)	315.5 (151.3–423)	0.767
Procalcitonin, M (IQR), ng/mL	0.687 (0.186–1.234)	8.72 (1.03–13.38)	0.0023

NEC, necrotizing enterocolitis; IQR, interquartile range; CGA, corrected gestational age.

## Data Availability

The fecal succinate data have been previously published [25]. Additional fecal metabolomic data derived from the same cohort were not included in the original publication and were reanalyzed in the present study to address a distinct scientific objective. The newly generated intestinal transcriptomic datasets generated and analyzed in this study have been deposited in the NCBI Sequence Read Archive (SRA) repository under BioProject accession number PRJNA1455810. All other data supporting the findings of this study are available within the article and its Supplementary Information files. Additional data are available from the corresponding authors upon reasonable request.

## Supplementary Materials

The following supporting information can be downloaded at: https://www.mdpi.com/article/10.3390/ijms27198691/s1.
