# Supplementary material for: Energy Metabolic Reprogramming Characterized by Succinate Accumulation Aggravates Necrotizing Enterocolitis
Source: Int J Mol Sci. 2026 Sep 29;27(19):8691. doi: 10.3390/ijms27198691 (PMC13665065; doi:10.3390/ijms27198691)
Supplement: Supplementary file 1 [file ijms-27-08691-s001.zip › Supplementary Materials.pdf]

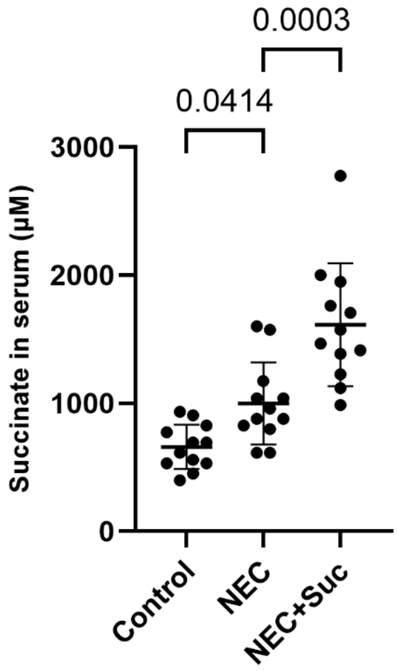


**Figure S1. Serum succinate levels in neonatal mice. Serum succinate concentrations were measured in Control, NEC, and NEC+Suc mice. Data are presented as mean ± SD (*n* = 12 per group). Statistical comparisons were performed using one-way ANOVA followed by Tukey's multiple-comparison test.**

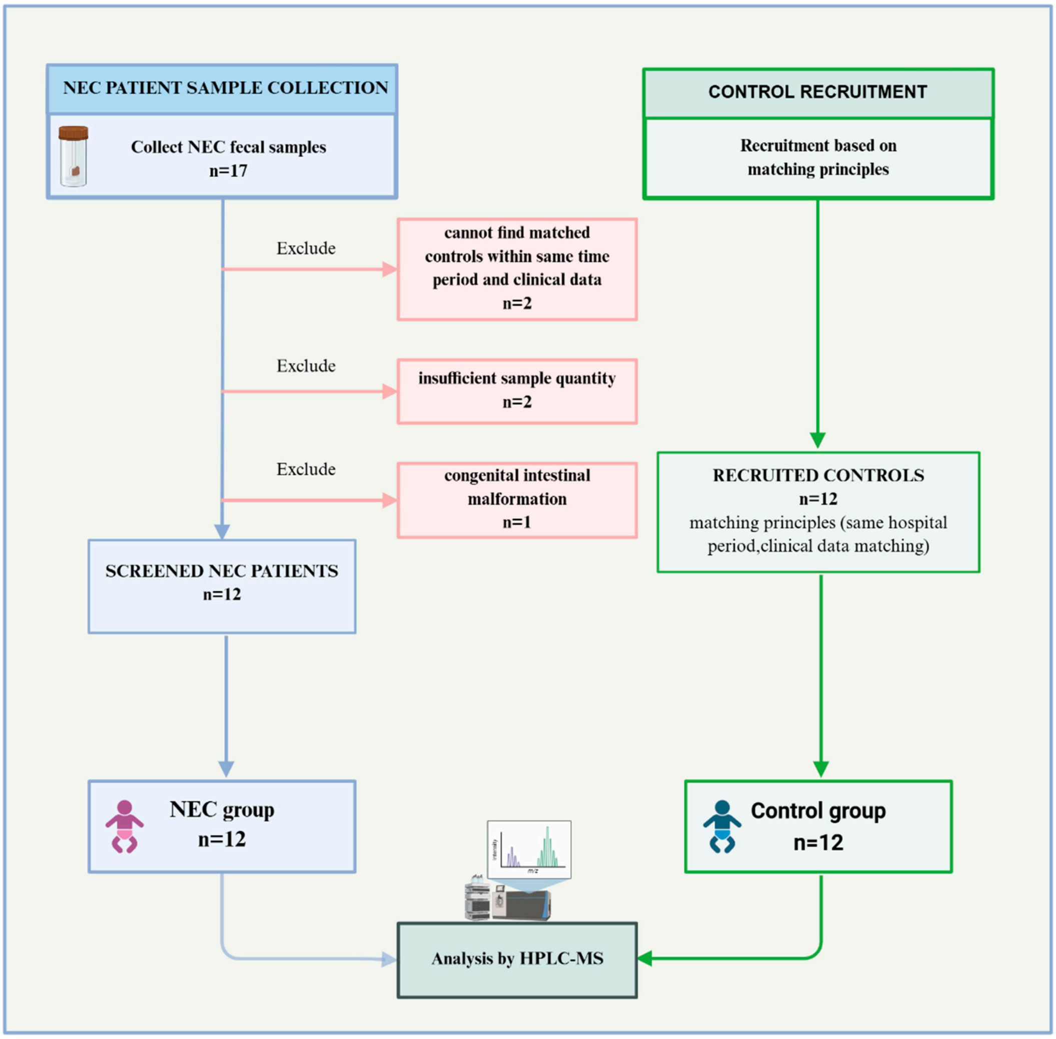


**Figure S2. Flowchart of the study design for exploring changes in fecal energy metabolites in preterm infants with NEC.**

The study flowchart was created using BioRender.com.

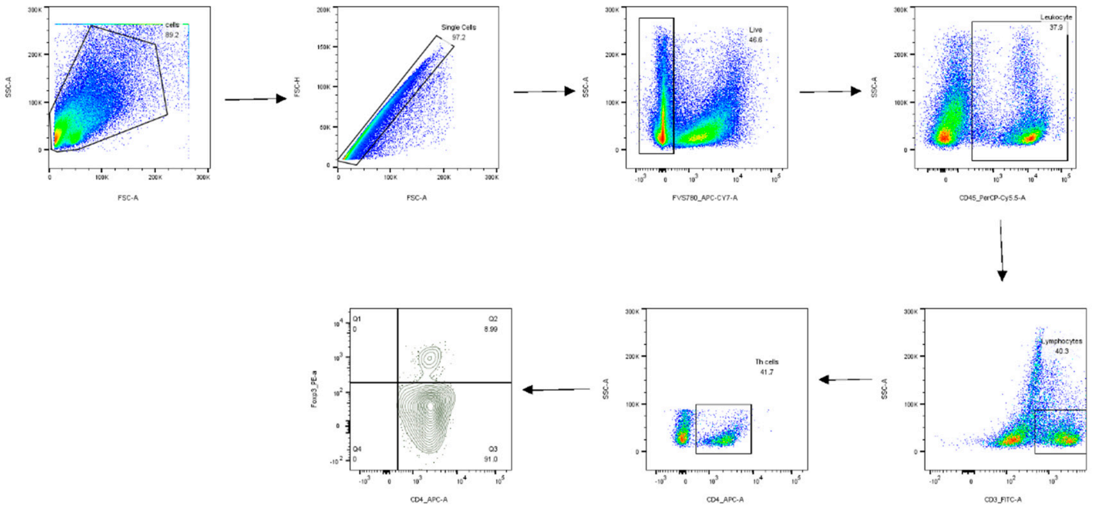


**Figure S3. Gating strategy for flow cytometric analysis of mouse intestinal tissue cells**
